# How much data do you need? An analysis of pelvic multi-organ segmentation in a limited data context

**DOI:** 10.1007/s13246-024-01514-w

**Published:** 2025-03-11

**Authors:** Febrio Lunardo, Laura Baker, Alex Tan, John Baines, Timothy Squire, Jason A. Dowling, Mostafa Rahimi Azghadi, Ashley G. Gillman

**Affiliations:** 1 Australian E-Health Research Centre, Commonwealth Scientific and Industrial Research Organisation, Surgical Treatment and Rehabilitation Service, 296 Herston Road, Brisbane, QLD 4029 Australia; 2 College of Science and Engineering, James Cook University, Townsville, QLD 4814 Australia; 3 Townsville Cancer Centre, The Townsville Hospital, Townsville, QLD 4814 Australia; 4 School of Medicine and Dentistry, James Cook University, Townsville, QLD 4814 Australia; 5 Sunshine Coast Hospital and Health Services, Sunshine Coast, QLD 4575 Australia

**Keywords:** Segmentation, Multi-organ segmentation, Prostate cancer, Seminal vesicles, Rectum, Bladder, Deep learning, MRI, Medical image, Training size

## Abstract

Training deep learning models generally requires large, costly datasets which can limit their application towards in-house segmentation tasks. This study investigates the trade-off in dataset size within the context of pelvic multi-organ MR segmentation where we evaluate the performance of nnU-Net, a well-known segmentation model, under conditions of limited domain and data availability. 12 participants undergoing treatment on an Elekta Unity were recruited, acquiring 58 MR images, with 4 participants (12 images) withheld for testing. Prostate, seminal vesicles (SV), bladder and rectum were contoured in each image by a radiation oncologist. Seven models were trained on progressively smaller subsets of the training dataset, simulating a limited dataset setting. To investigate the efficacy of data augmentation, another set of identical models were trained without augmentation. The performance of the networks was evaluated via the Dice Similarity Coefficient, mean surface distance, and 95% Hausdorff distance metrics. When trained with entire training dataset (46 images), the model achieved a mean Dice coefficient of 0.903 (Prostate), 0.851 (SV), 0.884 (Rectum) and 0.967 (Bladder). Segmentation performance remained stable when the number of training sets was > 12 images from 4 participants, but rapidly dropped in smaller data subsets. Data augmentation was found to be influential across all dataset sizes, but especially in very small datasets. This study demonstrated nnU-Net's proficiency in performing male pelvic multi-organ segmentation under a limited domain, a single scanner, and under limited data constraints. We found that the performance degradation was often modest until a threshold is reached (12 images), below which it dropped significantly. Data augmentation improved performance across all data sizes, but especially for very small datasets. We conclude that nnU-Net’s low data requirement can be advantageous for in-house cases with consistent protocol and scarce data availability.

**Supplementary Information:**

The online version contains supplementary material available at 10.1007/s13246-024-01514-w.

## Introduction

MRI-guided radiation therapy (MRgRT) is an established treatment option for prostate cancer [1, 2]. This treatment enables clinicians to perform real-time imaging with higher soft tissue contrast compared to CT-based treatment, and without additional use of ionising radiation. However, to fully realise these advantages, organs-at-risk (OAR) and target regions must be defined while the patient is on the couch so that the treatment plan can be adapted before delivery. Thus, fast and automatic, yet robust, segmentation techniques are particularly beneficial in the context of MRgRT.

There are extensive reports on automated pelvic organ segmentation [3]. While Convolutional Neural Network (CNN) based methods are popular, methods involving the application of transformers have recently been reported. Table 1 outlines state-of-the-art studies on pelvic organ MR segmentation. Most of these studies incorporate large public or private datasets, with minimal investigation done on the effects of small datasets.

**Table 1:** Previous approaches to pelvic organ segmentation (Note: performances are made on different datasets and direct comparisons should not be drawn)

Ref	First author surname	Total participants	Description	Performance (Dice)
1	Li et al. [4]	116 (train = 80, test = 36)	Developed low-cost auto segmentation method via semi supervised deep learning	Bladder: 0.954 (semi supervised) Rectum: 0.908 (semi supervised)
2	Ma et al. [5]	Public datasets (train = PROMISE12, test = NCI-ISBI13, Prostate158, PI-CAI)	Experimentation with using residual and gated connections into an encoder-decoder architecture	Prostate: 0.944
3	Vagni et al. [6]	60 (train = 40, val = 10, test = 10)	Implementation pf 3D GAN network to perform pelvic segmentation on 0.35 T MR-Linac. The 3D model’s performance on the internal test set is noted in this table	Bladder:0.83 (3D model version) Rectum: 0.92 (3D model version)
4	Kobayashi et al. [7]	93 (no details on split)	Applied 3d U-Net to segment prostate and surrounding extracapsular structures. The model’s mean performance on each organ with the verification dataset is noted in this table	Prostate: 0.83 Rectum: 0.78 Bladder: 0.83 Seminal Vesicles: 0.46
5	Lorenzen et al. [8]	68 participants (train = 38 (76 scans, test = 30(60scans)	Compared performance of nnU-Net against Clinical Deformable Image Registration algorithm (MONACO version 5.51.10). The model’s median performance on each organ with the test set is noted in this table	Prostate: 0.96 (nnU-Net) Rectum: 0.97 (nnU-Net) Bladder: 0.98 (nnU-Net) Seminal vesicles: 0.94 (nnU-Net)
6	Rodrigues et al. [3]	Public datasets (train = PROSTATEx, test = Medical Segmentation Decathlon)	Performed comparative study on 13 segmentation models. Found nnU-Net to be the best performing. The model’s performance on the external dataset for full volumes are presented in this table	Prostate (gland): 0.8678 (nnU-Net) Prostate (transition zone): 0.8760 (nnU-Net)
7	Wang et al. [9]	81 (no details on split)	Two stage CNN model (using Squeeze-Excitation module and Residual-Attention Unet (SERA))	Prostate: 0.860 (SERA)
8	Isaksson et al. [10]	100 scans (Did 2 different split ratio. Train/test = 70/30 and 50/50)	Comparison of multiple DL segmentation models against multi-atlas segmentation and proprietary segmentation algorithm	Prostate: 0.914 (EfficientDet)
9	DeSilvio et al.[11]	92 (train = 44, test = 48)	Assess the quality of region-specific U-Net models (to segment outer rectal wall, lumen, and perirectal fat regions) in a multi-institutional and multi inter-observer study	Outer rectal wall: 0.920 Lumen: 0.895
10	Ren et al. [12]	218 (train = 80%, val = 10%, test = 10%)	Experimentation with using transformer encoder in a multi-encoder and decoder segmentation network	Prostate: 0.95 (Muled-Net)
11	Yan et al. [13]	PROSTATEx (train = 160, test = 40) and Private set (in 3-Fold Cross Val)	Experimenting with a segmentation model that has characteristics of both CNN and transformer. Model performance on the private dataset is not noted in this table	Prostate (PZ): 0.8039 (PROSTATEx test set) Prostate (TZ): 0.8749 (PROSTATEx test set)
12	Vasconez et al. [14]	PROSTATEx (train = 120, test = 20)	Comparing segmentation performance of CNN vs Transformer based models. Also, tested influence of dataset size on model performance. Noted in this table is the model’s performance when trained with 120 images (the best performance)	Prostate: 0.87 (CNN based) and 0.86 (Transformer based)
13	Hyer et al. [15]	23 (fivefold, leave 20% out cross validation)	Investigate the use of automatic contouring algorithm to improve speed and reproducibility of contouring during MRgRT	Prostate: > 0.912 Rectum: > 0.912 Bladder: > 0.912 Seminal Vesicles: 0.842

Large and diverse datasets generally result in more robust and generalisable models [16–18]. However, the process of gathering and validating medical domain datasets incurs notable costs [19]. In situations where training data is limited, semi-supervised learning (SSL) techniques are often implemented [17, 18]. This type of learning encourages the model to incorporate unlabelled data in combination with the labelled data for training. Whilst some reports show semi-supervised models achieving similar results to top-performing fully supervised models [18], SSL models still exhibit disadvantages. These include potential bias introduced by class imbalance in their training dataset, the necessity to inspect the quality of unlabelled data, as low-quality unlabelled data can hurt overall performance, and added complexity/overhead to the model’s framework [18].

Another emerging technique gaining popularity is the Few Shot Learning (FSL). This method utilises a pre-trained model, originally trained on an unrelated task, and fine-tunes it only a few labelled samples (as low as 1) for the new task [20–22]. However, FSL also encounters challenges such as a lack of pre-trained models specifically for medical images [23] and cross-domain transferability issues (models must be pre-trained on a similar-enough task) [20, 24]. Motivated by these arguments and to limit this study’s scope, we explore the use of a small training dataset and the subsequent impacts on performance of a supervised model.

The model selected for this study is nnU-Net (version 2.2) [25], chosen due to its open-source availability, flexibility for modification [26] and demonstrated performance and robustness in prior literature [18, 27]. A review paper benchmarking the segmentation performance of U-Net variants found that nnU-Net outperformed other U-Net variants (i.e., Attention U-Nets, SegResNets, and U-Net + +) in Dice Similarity Coefficient (DSC) performance when applied on small datasets, less than 100 images [27]. Furthermore, a recent benchmark study showed that despite its earlier introduction, the nnU-Net framework still outperforms a more recently introduced framework, Auto3DSeg, which is part of the MONAI library [28].

There are potential applications for DL segmentation, for example for local, in-house contouring applications to accelerate specific workflows, where data may be scarce. In this study, we aimed to quantify the segmentation performance of nnU-Net trained with a limited training dataset condition. Seven different models were trained with each exposed to a progressively reduced training dataset. Finally, we presented a performance analysis on the models using an identical test set.

## Methodology

### Dataset

#### Dataset acquisition

With the approval of the Townsville Hospital and Health Service (THHS) Human Research Ethics Committee (reference number: HREC/QTHS/71867), images were obtained from consenting participants undergoing radiation therapy for prostate cancer at the THHS, Australia, between 2021 and 2024. The candidate’s inclusion criteria were histologically confirmed prostate cancer and aged 18 years or older. For each fraction, at least 4 MR images of the pelvic region were obtained for the study: a pre-treatment scan (before treatment replanning stage), a scan during treatment (replanning stage), a verification scan (before treatment beam delivery) and a post-treatment scan (after treatment beam delivery). All scans except those obtained during the treatment replanning stage were manually delineated (prostate, bladder, seminal vesicles (SVs), and rectum) by an experienced radiation therapist and radiation oncologist. Twelve participants, comprising a total of 58 images, were recruited, contoured and included in this study.

All images were volumetric-transverse T2 weighted images obtained on the Elekta Unity 1.5 T MR-Linac with the prostate located at the isocentre. Images were obtained with one of the two sets of scan parameters due to a change in site protocol, outlined in Table 2.

**Table 2:** Data scan parameters

	Scan parameters 1	Scan parameters 2
Scan Type	TSE T2 3D Tra	TSE T2 3D Tra
Reconstructed voxel size [mm]	0.833 × 0.833x1	0.833 × 0.833x1
Reconstructed matrix size	480 × 480x300	576 × 576x300
Repetition time [ms]	1535	1400
Echo time [ms]	277.818	182.726
Bandwidth (Hz)	740	744
Echo train length (TSE factor)	114	75
Number of scans	67	284

#### Limited training sets experiment

To observe the effect of training dataset size on model performance, 7 different models were trained using progressively smaller datasets. The reference model (Exp A) was trained with 46 labelled cases, and other models with nested subsets (Table 3). All models were tested on the same labelled test cohort consisting of 12 images from participants Pt9, Pt10, Pt11, Pt12.

**Table 3:** Train dataset variation table

Experiment	Total num of contoured images	No. unique participants
A (100%)	46	8 (Pt1-8)
B (87.5%)	40	8 (Pt1-8)
C (75%)	35	8 (Pt1-8)
D (50%)	23	7 (Pt1-7)
E (37.5%)	17	5 (Pt1-5)
F (25%)	12	4 (Pt1-4)
G (12.5%)	6	2 (Pt1-2)

To investigate the effect of data augmentation, training was performed with and without nnU-Net’s default augmentations (rotation, scaling, Gaussian noise, Gaussian blur, brightness, contrast, simulation of low resolution, gamma correction and mirroring [25]).

### Model and processing

MRI samples were pre-processed by resampling to a common reference and cropped to a field-of-view of 141 × 257 × 217 voxels that was chosen to include all ROIs in the dataset.

A nnU-Net models (version 2.2) [25] with the default model and training and inference pipelines (except for data augmentation in experiments omitting this), were trained for each experiment. The nnU-Net framework allows for automatic parameter configuration by examining the dataset’s characteristics. Fixed parameters such as optimizer and loss function, are consistent regardless of dataset. Rule-based parameters are dataset-dependent and include the image resampling strategy, patch size, batch size and network topology. Empirical parameters, which involve ensemble selection and the choice of post processing methodology, are chosen based on a trial-and-error method. Full details on how parameters for nnU-Net are generated can be found in its original paper. The optimised parameters are reported in Supplementary Materials 2. Training was conducted via fivefold cross validation. Model training and inference were conducted on a Tesla P100-SXM2 GPU with 16 GB of VRAM, 8 cores of the Dual Xeon 14-core E5-2690, and 32 GB of RAM.

In addition to nnU-Nets default postprocessing and to ensure connected masks were produced, only largest connected component was retained for bladder, rectum, and prostate. For SVs, the 2 largest components were retained to account for the left and right vesicles.

### Evaluation

The model’s segmentation performance was quantified with Dice Similarity Coefficient (DSC), 95% Hausdorff Distance (HD95) and Mean Surface Distance (MSD). DSC measures the overlap between ground truth and predicted organ masks, with scores ranging from 0 (no overlap) to 1 (perfect overlap), HD95 is calculated as the 95th percentile of the distances between boundary contours, and MSD measures the average distance between the boundaries of the contours.

Because the axial extent of contouring of the SVs and rectum were not specified in the contouring protocol, these varied significantly within the dataset. To exclude the uncertainty of axial contouring extent, the predicted contour for these organs was axially cropped to the axial field-of-view (FOV) of the ground truth.

## Results

The reference model’s performance can be seen in Table 4 and Fig. S1 in Supplementary Materials 1. These results demonstrate the model’s satisfactory performance. The model achieved a mean DSC score of at least 0.8 across all organs, and all organs except the rectum had an HD95 distance below 5 mm.

**Table 4:** Mean Dice, HD95, and MSD comparison against other state-of-the-art models

	DSC				95% hausdorff distance (mm)				Mean surface distance (mm)			
Models	Prostate	SV	Rectum	Bladder	Prostate	SV	Rectum	Bladder	Prostate	SV	Rectum	Bladder
ResGNet [5]	0.944	–	–	–	3.28	–	–	–	0.919	–	–	–
MONAI 3D U-Net [7]	0.83	0.46	0.78	0.83	4.4	7.4	5.3	5.8	–	–	–	–
Muled-Net [12]	**0.95**	–	–	–	9.56	–	–	–	**0.660**	–	–	–
Deep LOGISMOS [15]	> 0.912*	0.842*	> 0.912*	> 0.912*	2.5*	2.6*	2*	0.9*	-	–	–	–
3D GAN [6]	–	–	0.83	0.92	–	–	9.71	5.91	–	–	–	–
Semi Supervised 3D U-Net [4]	–	–	**0.918**	0.964	–	–	7.74	**0.605**	–	–	1.612	**0.017**
**Ours (Exp A)**	0.903	**0.851**	0.884	**0.967**	3.035	**2.454**	8.813	1.41	1.053	**0.683**	**1.081**	0.435

Values are as reported in paper on different/independent dataset and only provided as reference

*Median values, source paper did not provide mean

Bolded values represent the best performance

**Fig. 1:**
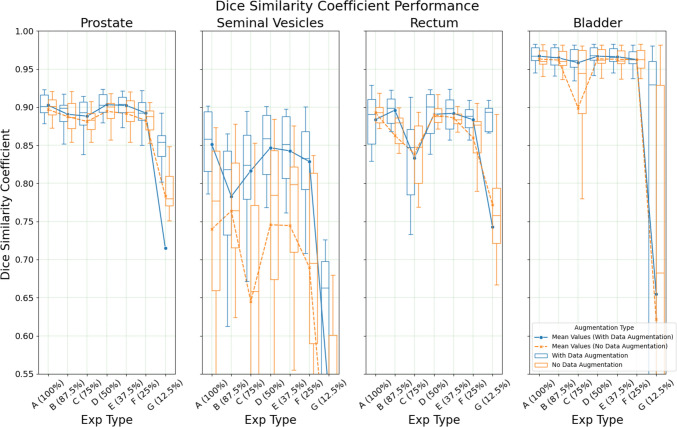
Organ DSC performance in a limited dataset setting with/out augmentation (Note: The plot domain is limited, resulting in clipping of some boxes)

DSC performance for each organ is depicted in Fig. 1, there is an improvement in DSC when the training data increases from 6 (Exp G) to 12 (Exp F) cases by 0.173, 0.090, 0.296, 0.303 in prostate, rectum, SV and bladder, respectively. However, beyond Exp F, improvements begin to plateau despite further increase in training size.

**Fig. 2:**
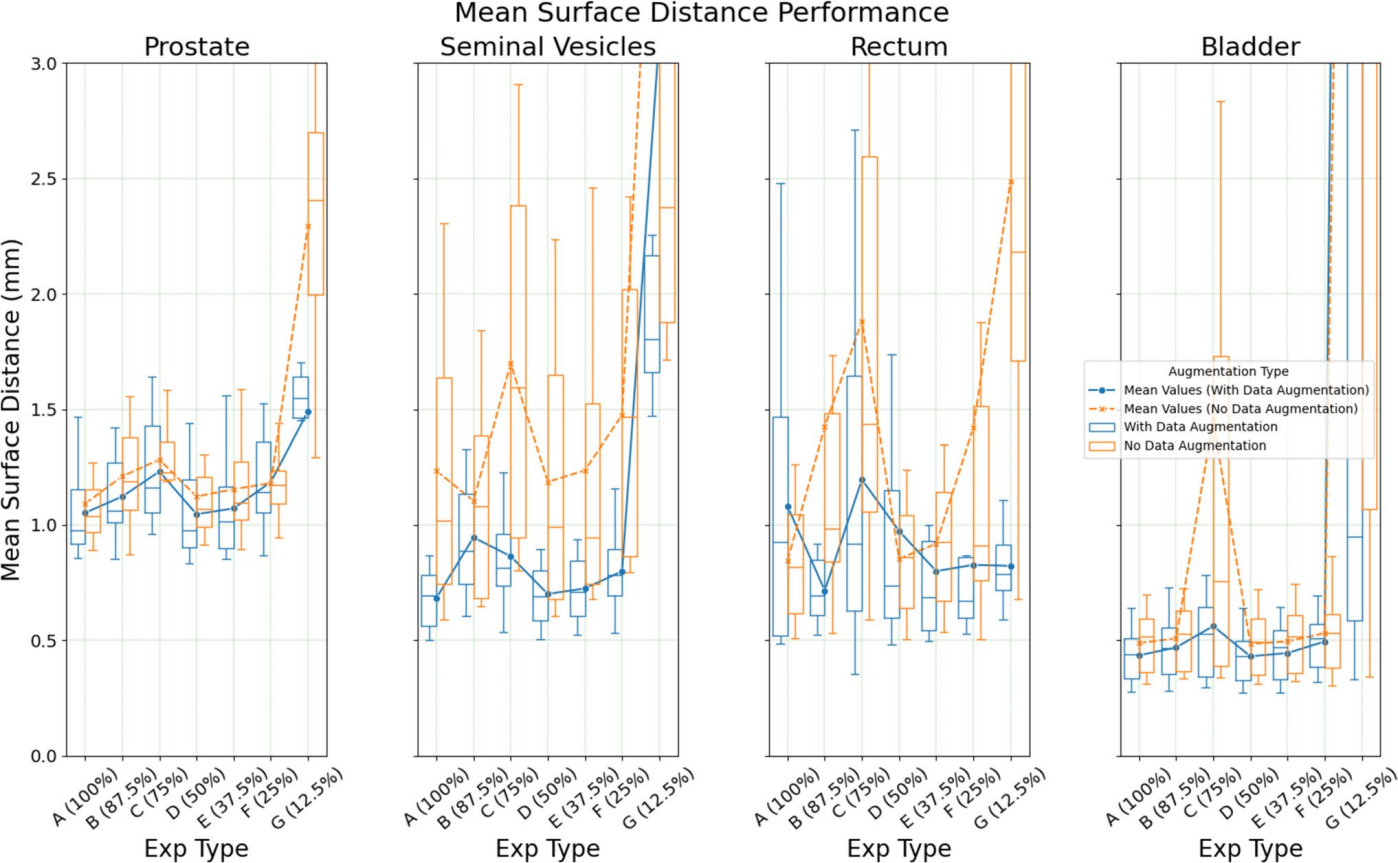
Organ MSD performance in a limited dataset setting with/out augmentation (Note: The plot domain is limited, resulting in clipping of some boxes)

**Fig. 3:**
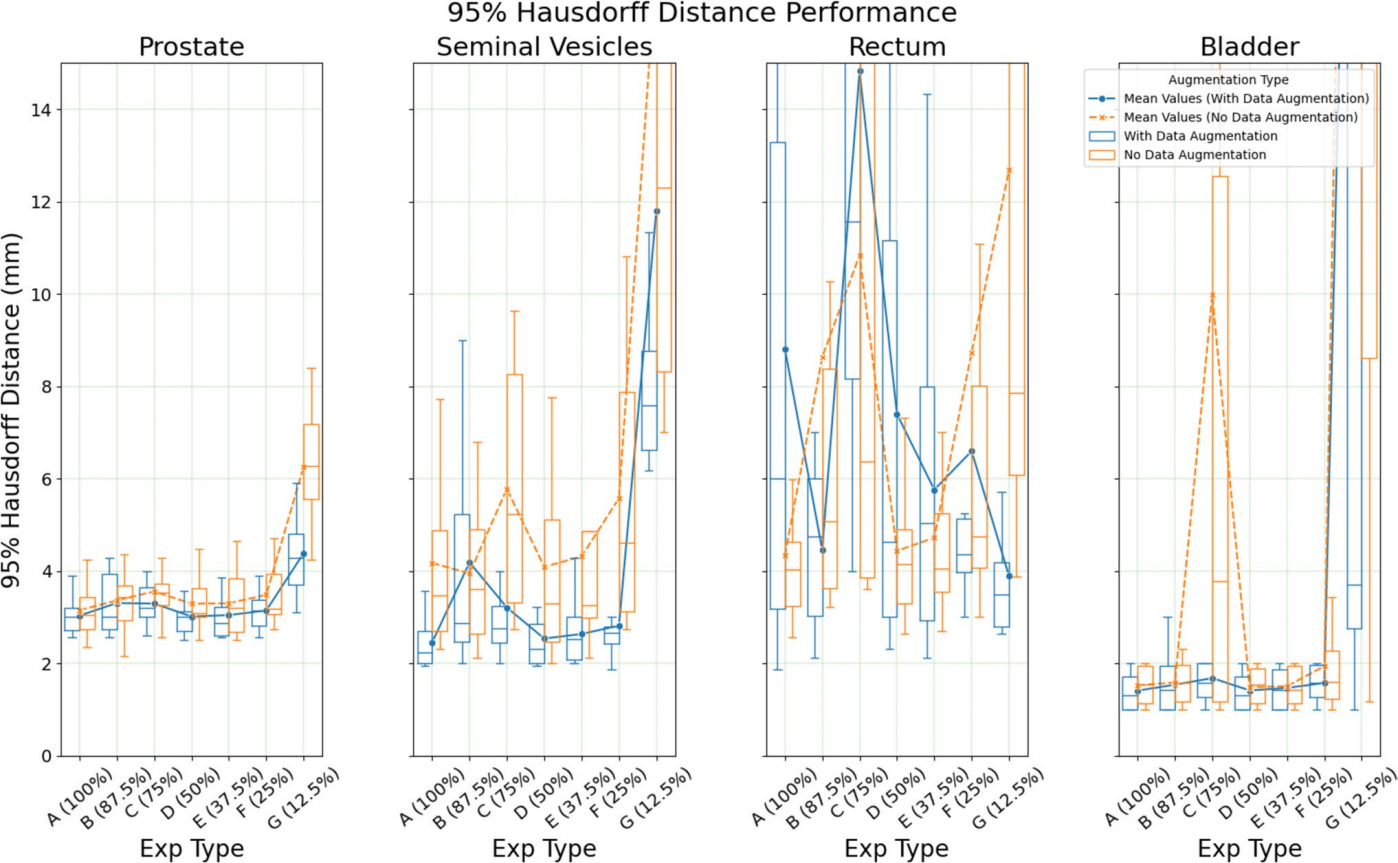
Organ HD95 performance in a limited dataset setting with/out Augmentation (Note: The plot domain is limited, resulting in clipping of some boxes)

There is a reduction in both MSD and HD95 performance as the training dataset is limited further (Figs. 2 and 3). The bladder and prostate experience incremental reductions in performance whilst maintaining a consistent range throughout multiple experiments. In contrast, the rectum and SV exhibit more unpredictable behaviour, where models trained with fewer data points perform better than those trained with more data points. This is seen in the rectum, where the model trained with 50% of the training dataset (Exp D) yielded a median HD95 of 4.752 and median MSD of 0.696 as opposed to model trained with 100% train data (Exp A) at a median HD95 of 6 and a mean MSD of 0.927.

Data augmentation generally benefits performance, particularly for models trained with the least data (i.e., Exp G in Fig. 1, 2, 3). Otherwise, the non-augmented models performed similarly to augmented models. Compared to the augmented counterparts, improvements in performance due to increases in dataset sizes are more significant. For example, between Exp A and Exp F, there was an improvement of 0.475 for prostate HD95 in the non-augmented compared to just 0.195 in the augmented models. Further details of the results are recorded in Tables S1 to S4 in the Supplementary Materials 1.

**Fig. 4:**
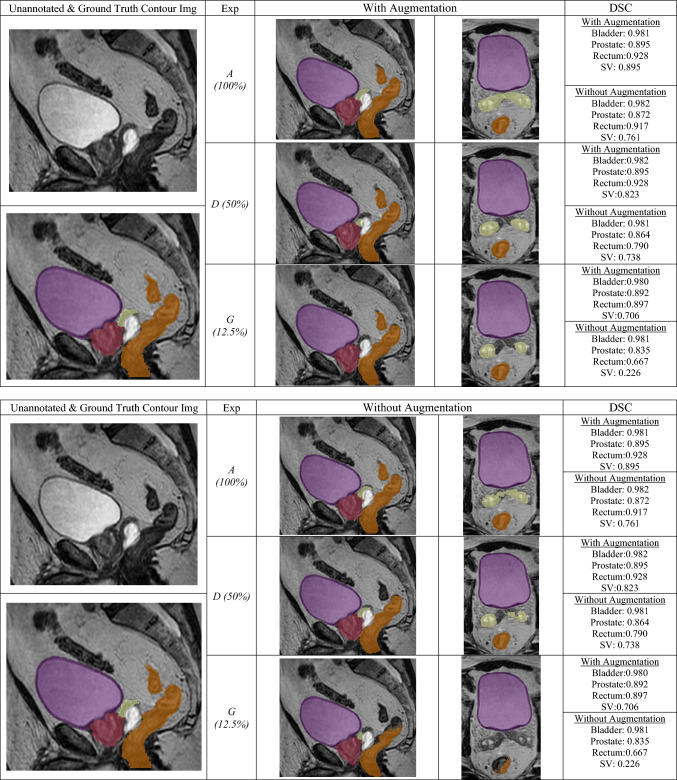
Model performance with/out augmentation of the same participant. bladder (purple), prostate (dark pink), rectum (brown), SV (yellow). This participant was selected for having the best average DSC across the four organs in the augmented model inference

Figure 4 depicts the visual performance of generated contours as the training data is further limited. The generated prostate and bladder contours remained relatively stable throughout, while the generated SV and rectum contours were significantly more affected as the training data was limited.

### Reference model performance in context of the state-of-the-art (SOTA) models in the literature

See Table 4.

#### Model performance in limited dataset setting (with/out influence of augmentation)

See Figs. 1, 2, 3, 4.

## Discussion

The purpose of this study was to explore the limits of nnU-Net under a limited training dataset. Previously, Bhandary et al. [27] found that in the context of a small training sample size (n < 100), nnU-Net outperformed other U-net variants. Similarly, by training an nnU-Net model with only 76 scans, Lorenzen et al. [8] were able to achieve a median DSC of 0.96 (prostate), 0.97 (rectum), 0.98 (bladder) and 0.94 (SVs) on the test set.

Results of this study further demonstrated nnU-Net's ability to perform pelvic multi organ segmentation with a limited dataset, showing that with a training dataset as small as 12–17 individual images from 4–5 participants can yield potentially acceptable results, 0.888/0.817/0.833/0.958 DSC for prostate/SV/rectum/bladder on the test cohort (Fig. 1) When comparing its DSC performance against other works nnU-Net still maintains comparable results against state-of-the-art models (Table 4). For prostate segmentation, nnU-Net achieved a DSC score of 0.903, while Muled-Net by Ren et al. [12]with a DSC score of 0.95. These results exceed the interobserver variability range of DSC, 0.88 ± 0.05, as reported by Roach et al. [29], indicating the reliability of the contours is within that expected of a human observer.

Increasing training data from 6 images from 2 participants (Exp G—12.5% train data) to 12 images from 4 participants (Exp F—25% train data) yielded the most noticeable improvement. However, beyond 12 training cases, only incremental improvements to the model performance were observed. This finding is consistent with other deep learning segmentation studies involving limited datasets. Vásconez et al. [14] examined the impact of reducing the training dataset on residual U-Net ‘s [10] prostate segmentation. The study observedthe most noticeable improvement when increasing the training datasets from 30 to 60 cases, with only marginal gains beyond that. Similarly, for prostate segmentation using a U-Net model, Bardis et al. [30] found that notable improvement was seen when training cases increased from 8 to 120. However, performance plateaued beyond 160 training cases.

One explanation for nnU-Net's robust performance could be the use of data augmentation during training [25]. Data augmentation is known to improve U-Net variant models’ performance trained on small datasets [31, 32] by artificially increasing the number of datapoints from existing samples. Additionally, other benefits of augmentations, such as reducing the risk of overfitting [16] and enhancing the model’s overall robustness against input variability [33, 34], have been well documented. This study supports these advantages conclusions, showing that augmentation improves performance, with a more pronounced effect in smaller datasets. Augmentation improved the segmentation of smaller or irregularly shaped organs, such as the SV and rectum, while it had smaller effects on larger organs (i.e. bladder and prostate). These results together demonstrate that the use of data augmentation is important in limited data contexts.

The following failure modes were most commonly noted when qualitatively assessing the segmentations. Firstly, due to similar intensities, the model incorrectly labelled hydrogel spacer as seminal vesicles and the pubic bone as bladder. This effect was more common in models trained with smaller training sets. Please refer to Figs. 1–10 in Supplementary Materials 3 for examples of these failure cases. Secondly, regardless of training size, the model was often inaccurate at the prostate/SV interface. Such inaccuracies are also observable in the ground truth labels, indicating that this variability may be learned by the models. Lastly, the contouring protocol did not explicitly define the superior-inferior borders extent of the SVs or rectum, meaning that the ground truth labels varied in axial coverage. To avoid penalising predictions that extended beyond the contoured axial extent for a given image, the prediction label was cropped to match the ground truth label’s extent during metric calculations.

This study evaluated nnU-Net on a relatively small testing cohort of 12 images, which may affect the generalisation of the results presented. To explore this limitation, we conducted an additional analysis on the model performance when performing inference on images independent to the testing set, concluding that the test performance tends to generalise over the acquired dataset, with some concerns about generalisation for the seminal vesicles and rectum at low data levels. These are unlikely to change the conclusions of our work. The details of this analysis can be found in Supplementary Materials 1 under ‘Generalisation Analysis’ section. Additionally, it’s important to note that data in this study only considered a limited domain context, namely a single Elekta Unity MR-Linac and only as applied to male pelvic anatomy. We did not explore the data requirements for segmentation in a heterogeneous domain context, explore any effects of domain generalisation to another scanner, nor non-pelvic anatomy. Lastly, we suspect our findings could be applicable to other body regions or MRI techniques. Our work has demonstrated that individual organs require varying levels of data to achieve reliable results. Conducting a separate investigation to determine the required training data sizes for other organs would be an interesting direction for future studies.

Domain generalisation to other scanners would be expected to be poor, but this could be acceptable for in-house applications with human supervision of automated algorithms. We also investigated only a small subset of possible convolutional architectures that nnU-Net explores, and no other models such as transformer-based models. These represent a point of interest for future research. Finally, nnU-Net currently incorporates a limited selection of augmentation methods. It would be valuable to explore the performance impact of techniques such as elastic deformation-based techniques [34], statistical shape methods [35, 36], GAN-based generative approaches to generate synthetic training data [37] or the utilisation of automatic augmentation strategy selection methods to identify optimal techniques for a specific dataset [38].

Leveraging the findings from this study and recognising the often-limited number of training datasets available in local medical physics or radiology departments, nnU-Net may serve as a valuable tool for clinical practitioners. Our work has demonstrated that, with a cohort of 48 images from 12 participants, nnU-Net can achieve segmentation accuracy beyond the reported interobserver variation. However, we have not yet established the model’s generalisation, reliability, or its robustness in a clinical environment. Despite these shortcomings, this technique’s minimal data requirement can be advantageous and useful in some low-risk, in-house contexts. For instance, models trained under such conditions may be useful in research studies where outliers may be acceptable. They can also be utilised to generate an initial contour for human-in-the-loop research pipelines, particularly in cases where commercial tools may not perform segmentation according to local site protocols.

## Conclusion

We assessed the performance of nnU-Net, an off-the-shelf, state-of-the-art segmentation network, in segmenting male pelvic organ anatomy. This study demonstrated nnU-Net's success in performing pelvic multi organ segmentation within limited datasets compared with the wider literature. Moreover, we found that the performance degradation as dataset size decreases was often modest until a threshold is reached (12 images), below which the performance dropped significantly. Data augmentation improved performance across all data sizes investigated, but especially for very small datasets.

## Supplementary Information

Below is the link to the electronic supplementary material.

Supplementary file1 (PDF 395 KB)

Supplementary file2 (XLSX 11 KB)

Supplementary file3 (PDF 1017 KB)

## Data Availability

Data is not available to the public due to ethics and privacy concerns.
