# Supplementary material for: How much data do you need? An analysis of pelvic multi-organ segmentation in a limited data context
Source: Phys Eng Sci Med. 2025 Mar 11;48(1):409–19. doi: 10.1007/s13246-024-01514-w (PMC11996946; doi:10.1007/s13246-024-01514-w)
Supplement: Supplementary file 1 — Supplementary file1 (PDF 395 KB) [file 13246_2024_1514_MOESM1_ESM.pdf]

Supplementary Materials 1

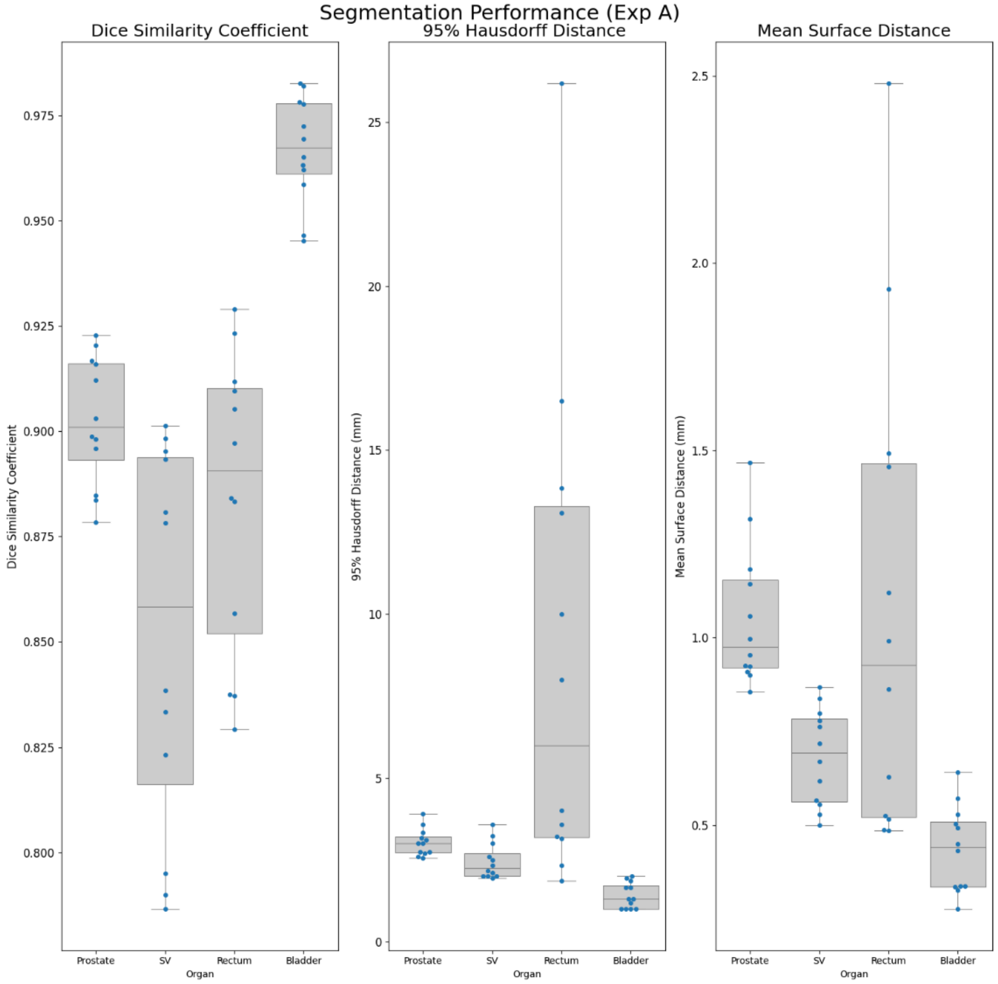

Fig. S1 DSC, 95% Hausdorff Distance and Mean Surface Distance Results of the Reference Model (Exp A) on Labelled Test Data

Prostate

	DSC		95% Hausdorff Distance (mm)		Mean Surface Distance (mm)	
**Exp Type**	**With Augmentation**	**No Augmentation**	**With Augmentation**	**No Augmentation**	**With Augmentation**	**No Augmentation**
A (100%)	0.903 [0.893 - 0.916]	0.897 [0.890 - 0.910]	3.035 [2.723 - 3.208]	3.152 [2.734 - 3.432]	1.053 [0.920 - 1.154]	1.092 [0.968 - 1.154]
B (87.5%)	0.903 [0.894 - 0.917]	0.894 [0.885 - 0.905]	3.015 [2.693 - 3.119]	3.293 [3.000 - 3.621]	1.047 [0.902 - 1.197]	1.124 [0.994 - 1.210]
C (75%)	0.902 [0.893 - 0.913]	0.892 [0.881 - 0.908]	3.047 [2.603 - 3.228]	3.303 [2.678 - 3.833]	1.072 [0.898 - 1.165]	1.155 [1.022 - 1.275]
D (50%)	0.891 [0.881 - 0.903]	0.888 [0.872 - 0.905]	3.307 [2.734 - 3.929]	3.364 [2.923 - 3.679]	1.123 [1.011 - 1.271]	1.211 [1.067 - 1.380]
E (37.5%)	0.892 [0.883 - 0.906]	0.883 [0.870 - 0.895]	3.146 [2.819 - 3.383]	3.483 [3.070 - 3.936]	1.186 [1.055 - 1.360]	1.182 [1.093 - 1.237]
F (25%)	0.888 [0.877 - 0.907]	0.881 [0.871 - 0.892]	3.296 [3.003 - 3.639]	3.558 [3.260 - 3.727]	1.232 [1.054 - 1.430]	1.283 [1.197 - 1.361]
G (12.5%)	0.715 [0.835 - 0.863]	0.783 [0.771 - 0.809]	4.386 [3.713 - 4.798]	6.261 [5.555 - 7.181]	1.492 [1.464 - 1.643]	2.293 [1.998 - 2.699]

Table S2 Prostate Segmentation Performance in Limited Dataset Setting. The metrics are presented as the mean [25th-75th percentile]. Note: * means these predictions included null predictions, resulting in some incalculable distance metrics. Such predictions were omitted, meaning that reported results are biased by omitting the worst predictions.

Seminal Vesicles

	DSC		95% Hausdorff Distance (mm)		Mean Surface Distance (mm)	
**Exp Type**	**With Augmentation**	**No Augmentation**	**With Augmentation**	**No Augmentation**	**With Augmentation**	**No Augmentation**
A (100%)	0.851 [0.816 - 0.894]	0.740 [0.659 - 0.843]	2.454 [2.000 - 2.703]	4.175 [2.701 - 4.877]	0.683 [0.563 - 0.784]	1.235 [0.744 - 1.636]
B (87.5%)	0.847 [0.812 - 0.889]	0.746 [0.674 - 0.843]	2.539 [2.000 - 2.864]	4.089 [2.464 - 5.107]	0.701 [0.587 - 0.801]	1.187 [0.681 - 1.648]
C (75%)	0.843 [0.807 - 0.888]	0.745 [0.710 - 0.822]	2.637 [2.086 - 3.000]	4.316 [2.994 - 4.870]	0.725 [0.607 - 0.844]	1.237 [0.744 - 1.527]
D (50%)	0.783 [0.732 - 0.843]	0.764 [0.715 - 0.827]	4.187 [2.464 - 5.226]	3.947 [2.639 - 4.894]	0.946 [0.745 - 1.135]	1.104 [0.683 - 1.387]
E (37.5%)	0.829 [0.792 - 0.868]	0.690 [0.590 - 0.813]	2.818 [2.420 - 2.801]	5.574 [3.125 - 7.869]	0.799 [0.696 - 0.895]	1.476 [0.866 - 2.021]
F (25%)	0.817 [0.780 - 0.863]	0.645 [0.486 - 0.771]	3.211 [2.453 - 3.250]	5.781 [3.327 - 8.258]	0.865 [0.735 - 0.962]	1.698 [0.947 - 2.383]
G (12.5%)	0.521 [0.407 - 0.697]	0.396 [0.215 - 0.601]	11.802 [6.624 - 8.757]	17.202 [8.328 - 22.637]	3.115 [1.659 - 2.167]	4.574 [1.878 - 6.946]

Table S3 SV Segmentation Performance in Limited Dataset Setting. The metrics are presented as the mean [25th-75th percentile]. Note: * means these predictions included null predictions, resulting in some incalculable distance metrics. Such predictions were omitted, meaning that reported results are biased by omitting the worst predictions.

Rectum

	DSC		95% Hausdorff Distance (mm)		Mean Surface Distance (mm)	
**Exp Type**	**With Augmentation**	**No Augmentation**	**With Augmentation**	**No Augmentation**	**With Augmentation**	**No Augmentation**
A (100%)	0.884 [0.852 - 0.910]	0.893 [0.880 - 0.900]	8.813 [3.191 - 13.284]	4.341 [3.250 - 4.632]	1.081 [0.521 - 1.466]	0.846 [0.616 - 1.047]
B (87.5%)	0.891 [0.866 - 0.916]	0.890 [0.880 - 0.898]	7.402 [3.002 - 11.164]	4.436 [3.300 - 4.910]	0.974 [0.600 - 1.150]	0.854 [0.639 - 1.042]
C (75%)	0.892 [0.871 - 0.910]	0.886 [0.879 - 0.891]	5.761 [2.924 - 8.000]	4.723 [3.556 - 5.250]	0.800 [0.543 - 0.931]	0.918 [0.670 - 1.143]
D (50%)	0.896 [0.879 - 0.911]	0.863 [0.852 - 0.886]	4.460 [3.026 - 6.000]	8.633 [3.626 - 8.383]	0.716 [0.609 - 0.848]	1.425 [0.840 - 1.482]
E (37.5%)	0.884 [0.873 - 0.897]	0.860 [0.840 - 0.881]	6.597 [3.976 - 5.138]	8.731 [4.064 - 8.020]	0.827 [0.598 - 0.861]	1.422 [0.760 - 1.514]
F (25%)	0.833 [0.785 - 0.871]	0.839 [0.800 - 0.876]	14.826 [8.169 - 21.734]	10.840 [3.859 - 15.187]	1.197 [0.628 - 1.647]	1.883 [1.059 - 2.595]
G (12.5%)	0.743 [0.868 - 0.899]	0.772 [0.722 - 0.794]	3.891 [2.800 - 4.181]	12.683 [6.085 - 17.384]	0.823 [0.717 - 0.915]	2.487 [1.710 - 3.066]

Table S4 Rectum Segmentation Performance in Limited Dataset Setting. The metrics are presented as the mean [25th-75th percentile]. Note: * means these predictions included null predictions, resulting in some incalculable distance metrics. Such predictions were omitted, meaning that reported results are biased by omitting the worst predictions.

Bladder

	DSC		95% Hausdorff Distance (mm)		Mean Surface Distance (mm)	
**Exp Type**	**With Augmentation**	**No Augmentation**	**With Augmentation**	**No Augmentation**	**With Augmentation**	**No Augmentation**
A (100%)	0.967 [0.961 - 0.978]	0.963 [0.957 - 0.974]	1.410 [1.000 - 1.716]	1.527 [1.134 - 1.944]	0.435 [0.336 - 0.509]	0.488 [0.363 - 0.595]
B (87.5%)	0.967 [0.962 - 0.978]	0.963 [0.958 - 0.976]	1.415 [1.000 - 1.716]	1.483 [1.134 - 1.883]	0.431 [0.326 - 0.499]	0.485 [0.353 - 0.595]
C (75%)	0.966 [0.960 - 0.977]	0.963 [0.957 - 0.974]	1.468 [1.000 - 1.863]	1.507 [1.134 - 1.944]	0.445 [0.333 - 0.542]	0.496 [0.360 - 0.610]
D (50%)	0.965 [0.956 - 0.978]	0.962 [0.955 - 0.973]	1.538 [1.000 - 1.944]	1.586 [1.179 - 1.958]	0.469 [0.355 - 0.556]	0.509 [0.366 - 0.631]
E (37.5%)	0.962 [0.957 - 0.973]	0.962 [0.952 - 0.975]	1.578 [1.271 - 1.958]	1.937 [1.226 - 2.273]	0.495 [0.384 - 0.572]	0.532 [0.380 - 0.614]
F (25%)	0.958 [0.953 - 0.976]	0.899 [0.892 - 0.975]	1.682 [1.271 - 2.000]	9.977 [1.179 - 12.544]	0.562 [0.343 - 0.643]	1.453 [0.390 - 1.731]
G (12.5%)	0.655 [0.338 - 0.960]	0.622 [0.477 - 0.929]	35.532 [2.750 - 76.173]	45.037 [8.613 - 76.532]	16.104 [0.586 - 13.260]	10.788 [1.069 - 9.351]

Table S5 Bladder Segmentation Performance in Limited Dataset Setting. The metrics are presented as the mean [25th-75th percentile]. Note: * means these predictions included null predictions, resulting in some incalculable distance metrics. Such predictions were omitted, meaning that reported results are biased by omitting the worst prediction

Generalisation Analysis

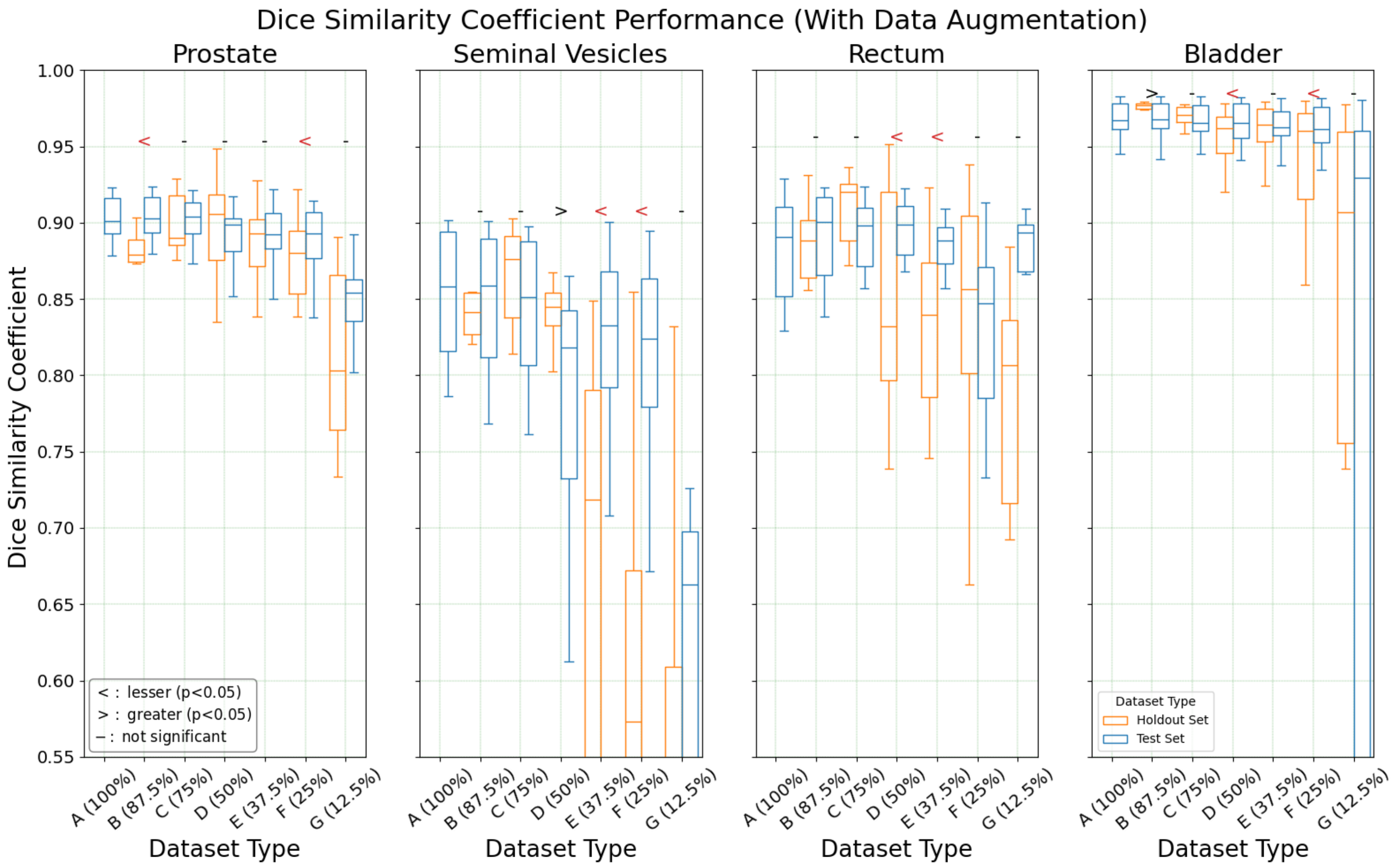

Fig. S6 DSC of the Generalisation Analysis on All Models. The <, > and – symbols within these graphs indicate whether mean performance on the holdout set and the test set are statistically less, greater, or not significantly different, respectively.

A relatively small testing dataset of 12 images from 4 participants was used to assess the models' performance. To better assess the measured performance generalisation over the larger acquired dataset, we compared the performance on the test set with the performance on held-out data in experiments that did not leverage the full training dataset (i.e., in the restricted dataset analysis). The size of the holdout set increased as more training data was withheld during model training (Table S7). We aim to demonstrate that the performance on the holdout set is not inferior to that of the test set (i.e. to demonstrate that the test set performance generalises over the acquired dataset).

The result of this investigation is presented in Fig S6. In most cases, the holdout set performance was not inferior to the test set performance, although slightly more cases were significantly inferior compared to significantly superior, indicating a measurable generalisation error. The mean difference per region was 0.0019/0.0816/0.0387/0.0130 for Prostate, SV, Rectum and Bladder, respectively, providing an approximate estimate of the generalisation error over the holdout set.

For the seminal vesicles and rectum, the relative performance on the holdout set deteriorates as the training size decreases. This suggests that generalisation error may be more significant, and the reported performance may be overestimated for more complex and small ROIs at low training data levels. However, this would not invalidate the conclusions of the work, and the training size thresholds for performance stability are unaffected.

Experiment	Total Num of Holdout Set Images
A (100%)	0
B (87.5%)	6
C (75%)	11
D (50%)	23
E (37.5%)	29
F (25%)	34
G (12.5%)	40

Table S7 Holdout Set Sizes
