# Supplementary material for: How much data do you need? An analysis of pelvic multi-organ segmentation in a limited data context
Source: Phys Eng Sci Med. 2025 Mar 11;48(1):409–19. doi: 10.1007/s13246-024-01514-w (PMC11996946; doi:10.1007/s13246-024-01514-w)
Supplement: Supplementary file 3 — Supplementary file3 (PDF 1017 KB) [file 13246_2024_1514_MOESM3_ESM.pdf]

Supplementary Materials 3 (Other Failure Cases)

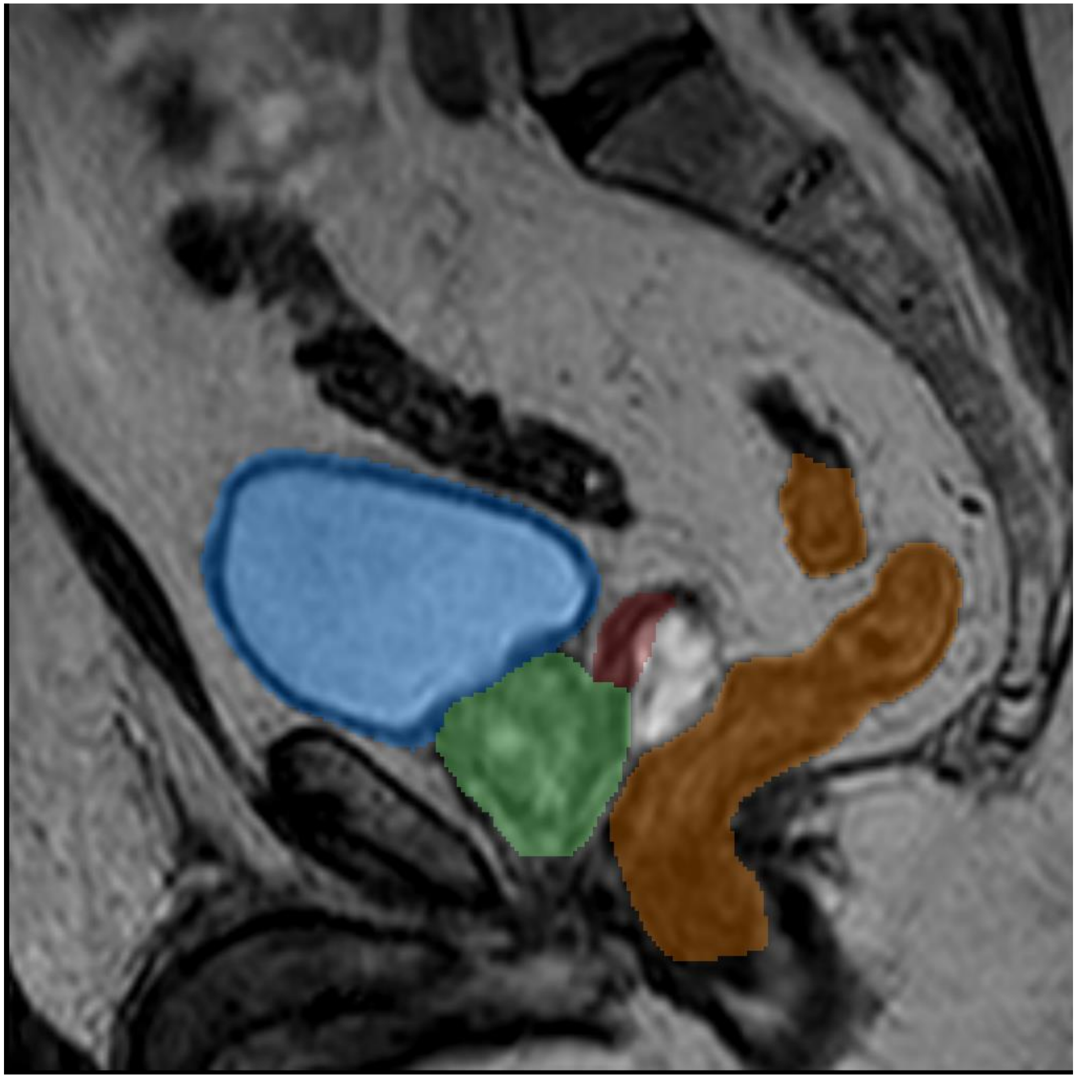

Fig 1: Sagittal View of a Case where Hydrogel was erronpaeously identified as SV

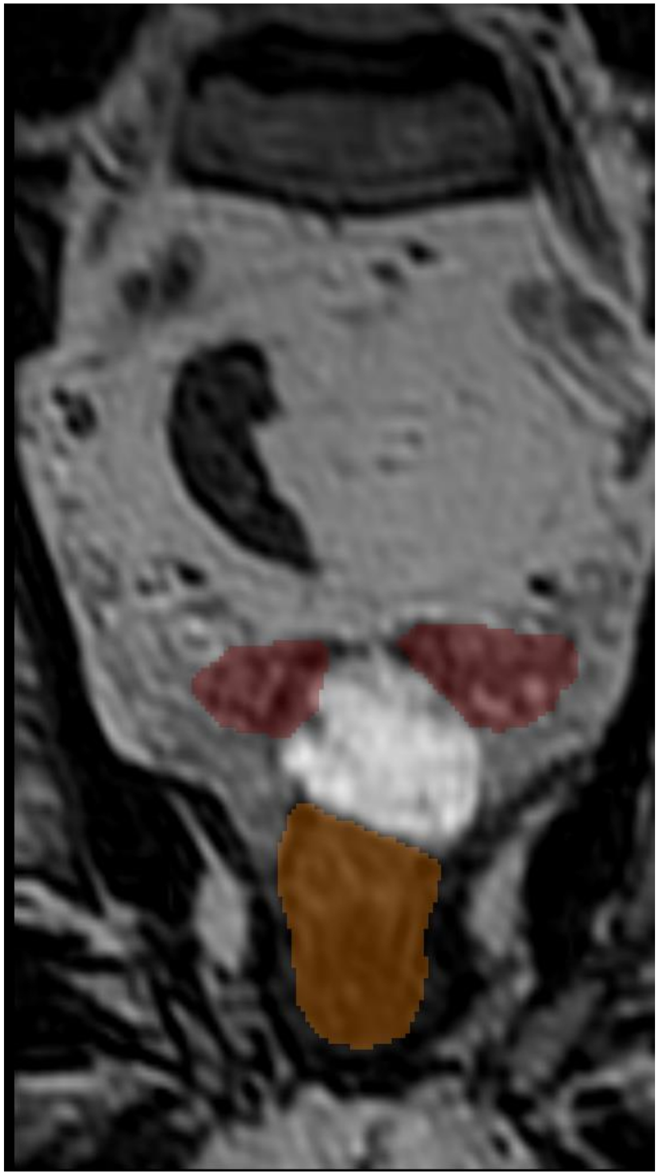

Fig 2: Coronal View of a Case where Hydrogel was erroneously identified as SV

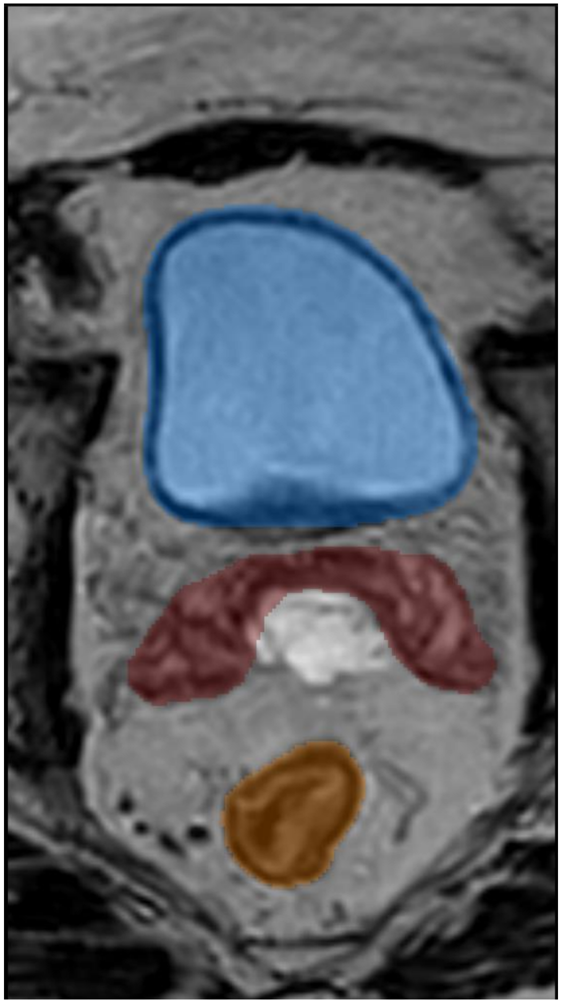

Fig 3: Axial View of a Case where Hydrogel erroneously identified as SV

Fig 1- 3 are examples of cases where the model incorrectly labelled hydrogel as seminal vesicles. These images were the inference output of a model trained with 35 images (Exp C).

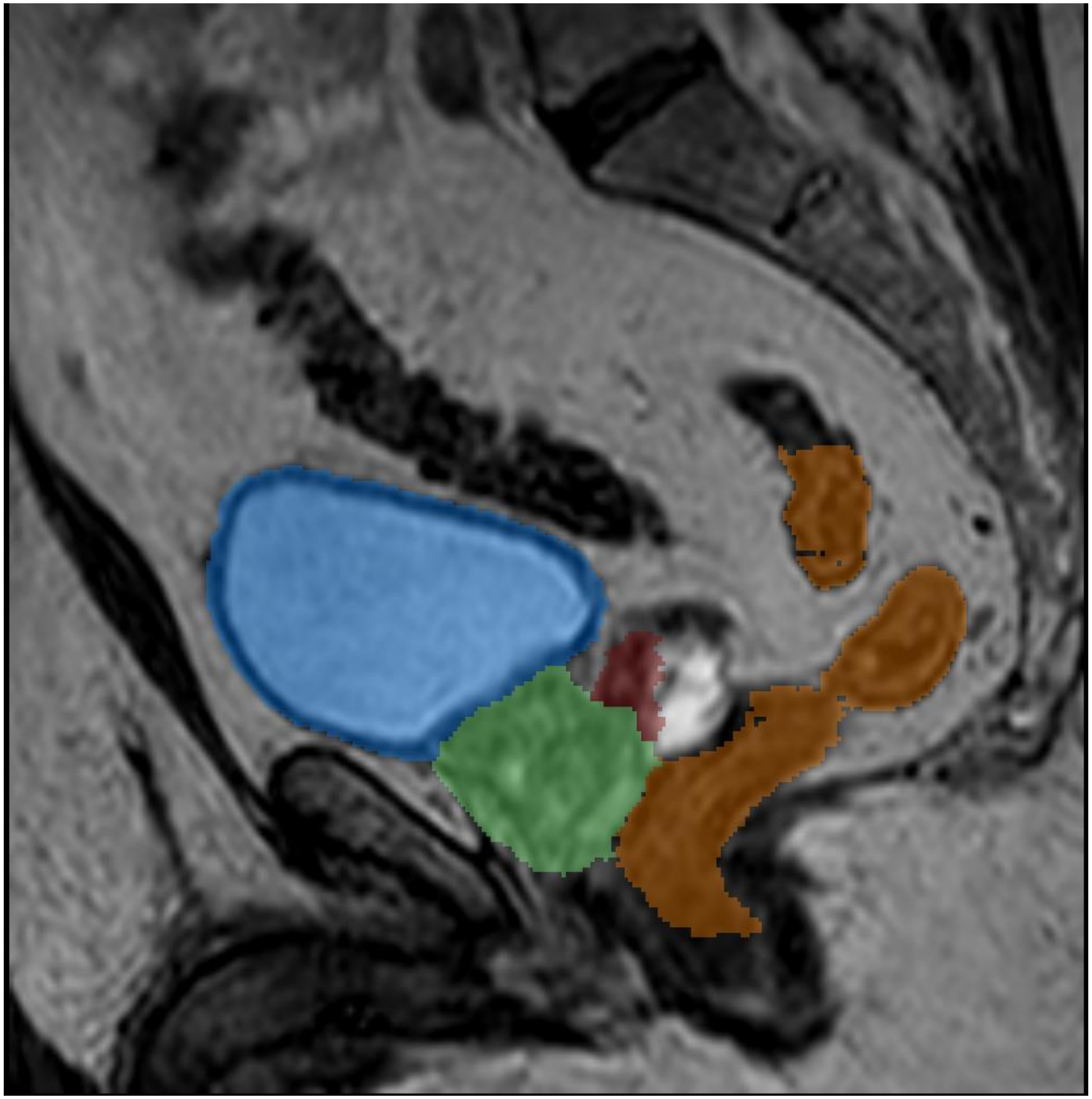

Fig 4: Sagittal View of a Case where Hydrogel was erroneously identified as SV

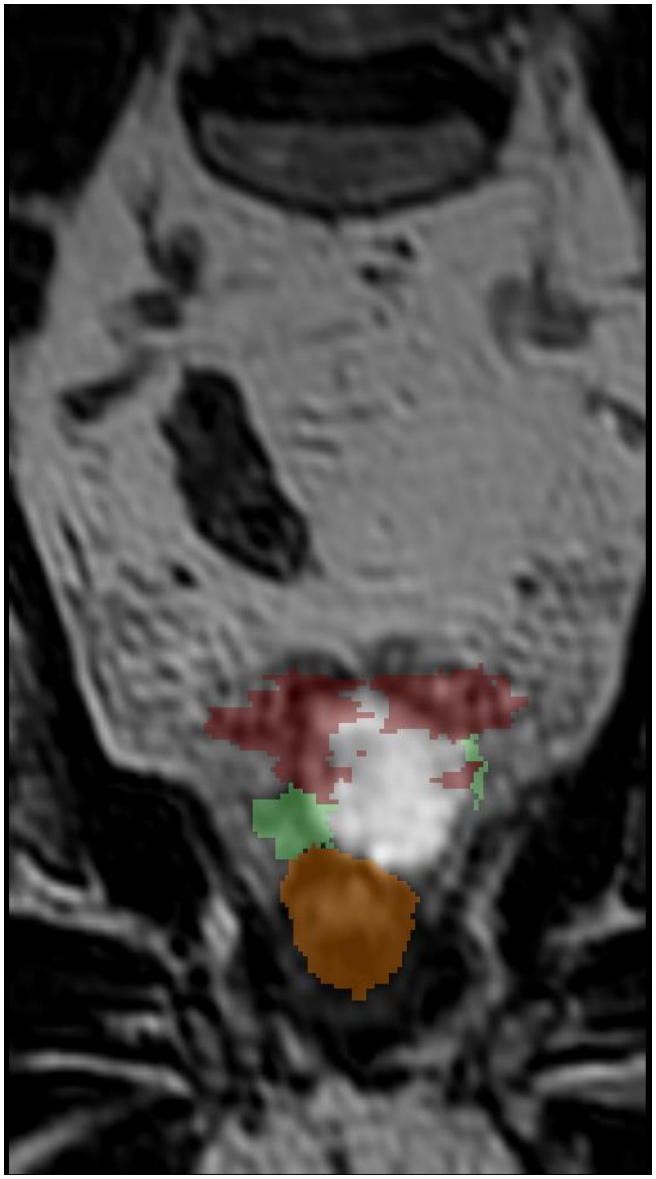

Fig 5: Coronal View of a Case where Hydrogel was erroneously identified as SV

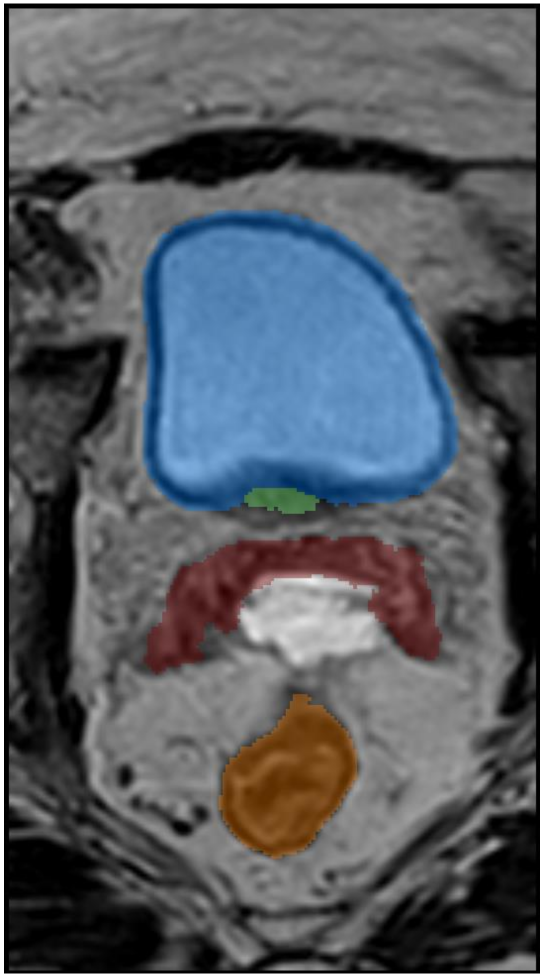

Fig 6: Axial View of a Case where Hydrogel was erroneously identified as SV

Fig 4- 6 are examples of cases where the model incorrectly labelled hydrogel as seminal vesicles. These images were the inference output of a model trained with 17 images (Exp E).

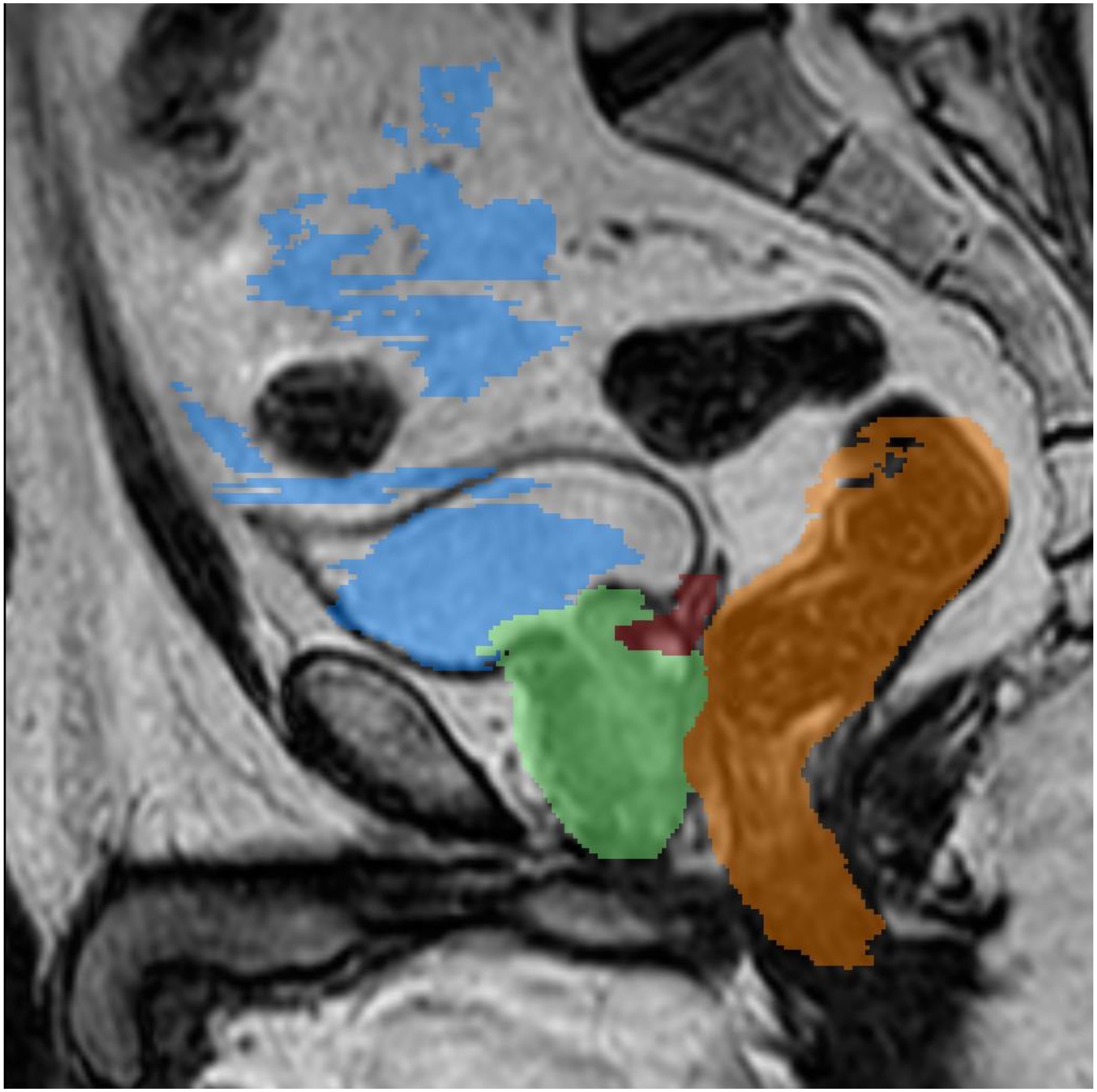

Fig 7: Sagittal View of a Case where bladder, rectum and the prostate/SV interface were poorly contoured

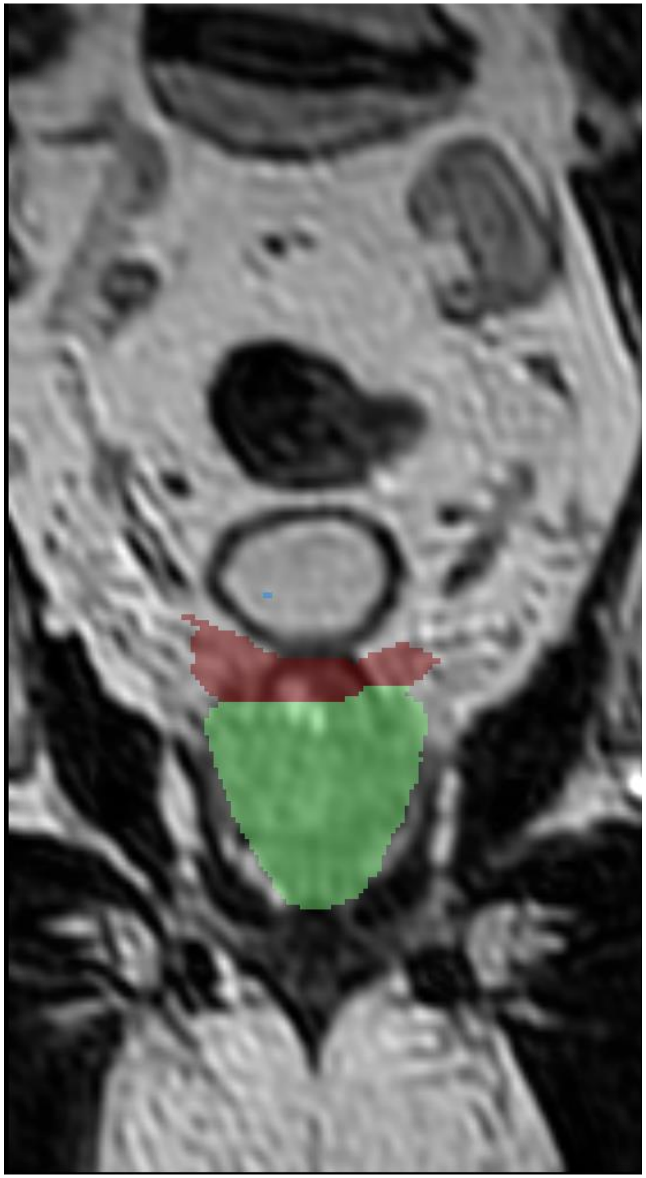

Fig 8: Coronal View of a Case where bladder, rectum and the prostate/SV interface were poorly contoured

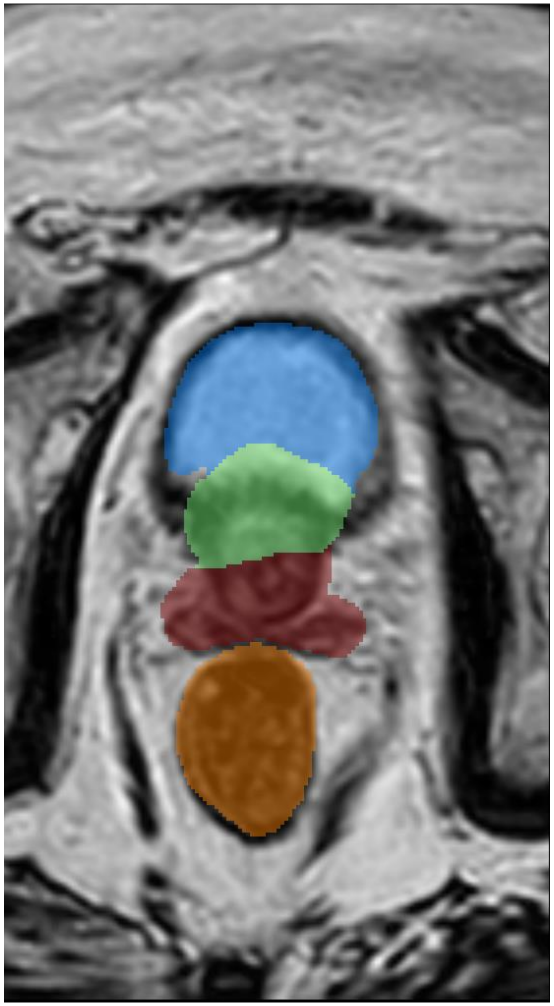

Fig 9: Axial View of a Case where bladder, rectum and the prostate/SV interface were poorly contoured

Fig 7- 9 are examples of cases where the model poorly labelled the bladder, rectum and the prostate/SV interface. These images were the inference output of a model trained with 6 images (Exp G).

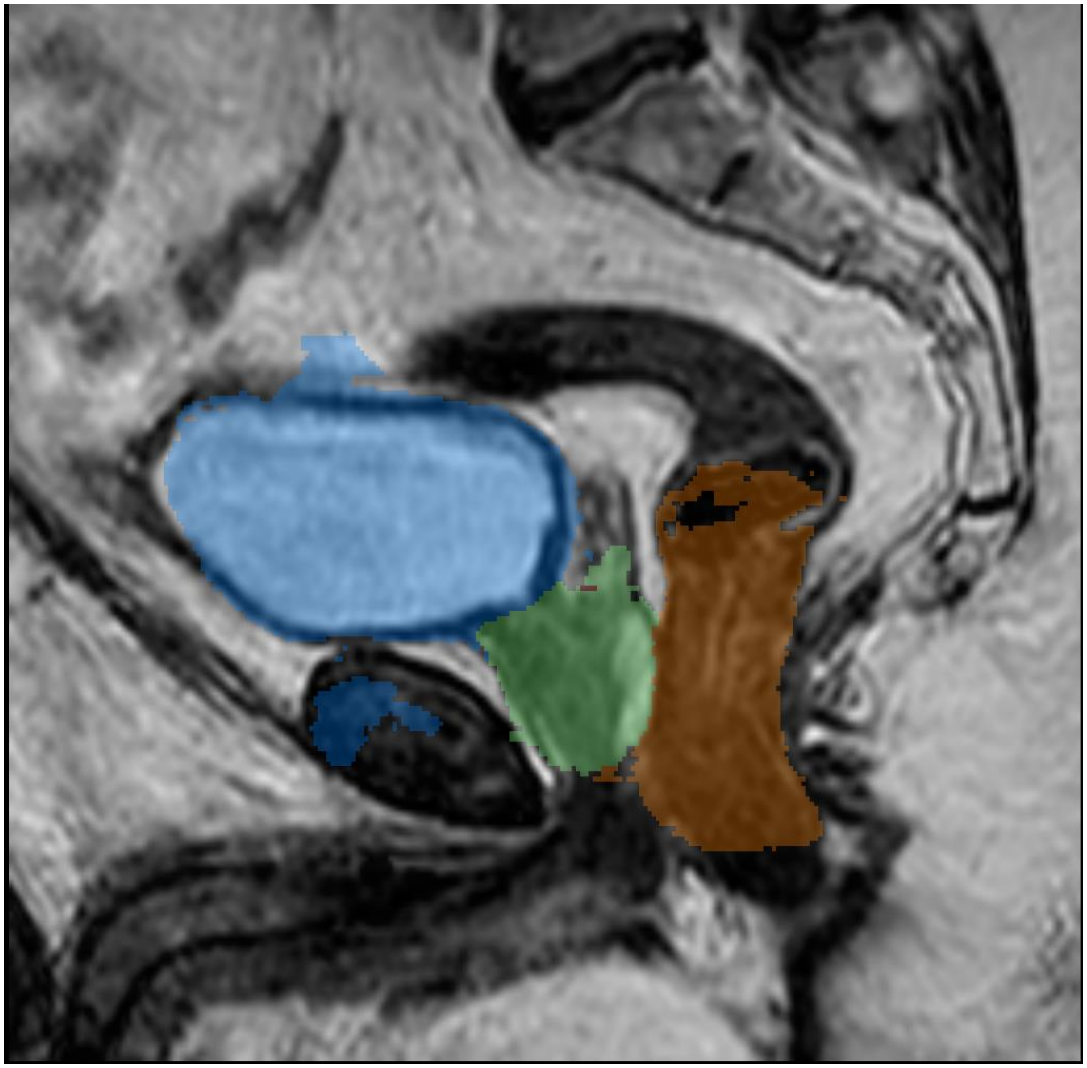

Fig 10: Sagittal View of a Case where pubic bone is labelled as bladder

Fig 10 is a case example where the model incorrectly labelled the pubic bone as bladder. Additionally, SV is not labelled. This image was the inference output of a model trained with 12images (Exp F).

Unannotated & Ground Truth Contour Img	Exp	With Augmentation		DSC
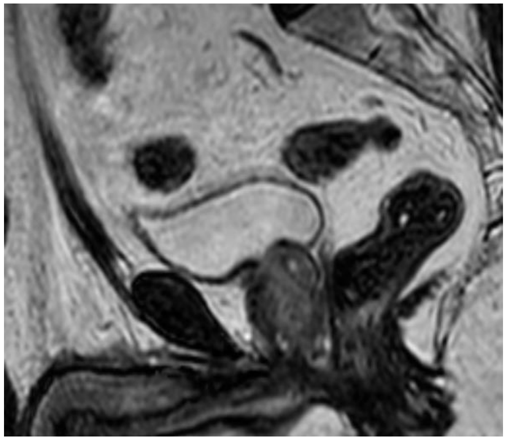	*A*<br>*(100%)*	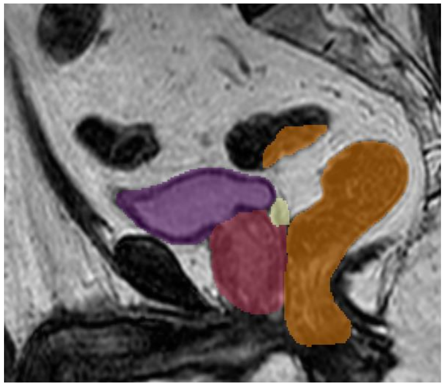	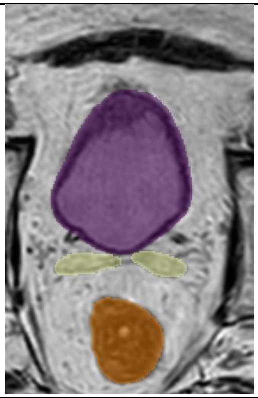	<u>With Augmentation</u><br>Bladder: 0.945<br>Prostate: 0.878<br>Rectum:0.837<br>SV: 0.823<br><u>Without Augmentation</u><br>Bladder: 0.940<br>Prostate: 0.856<br>Rectum:0. 880<br>SV: 0. 837
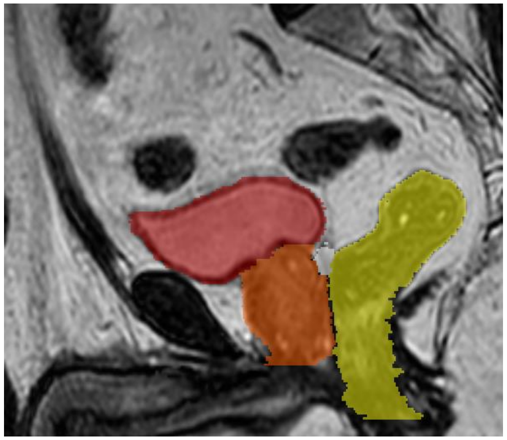	*D*<br>*(50%)*	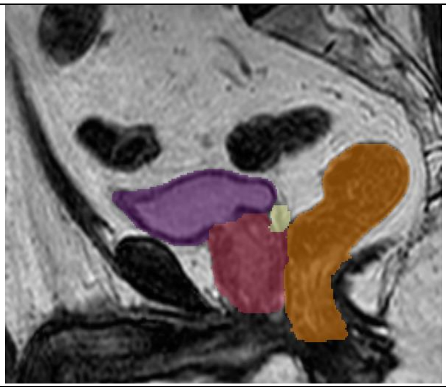	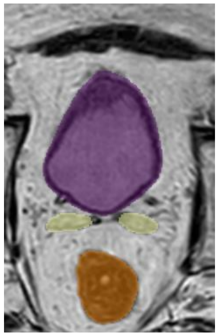	<u>With Augmentation</u><br>Bladder:0.945<br>Prostate:0.862<br>Rectum:0.915<br>SV:0.812<br><u>Without Augmentation</u><br>Bladder: 0. 941<br>Prostate: 0.857<br>Rectum:0.839<br>SV: 0. 812
	G<br>*(12.5%)*	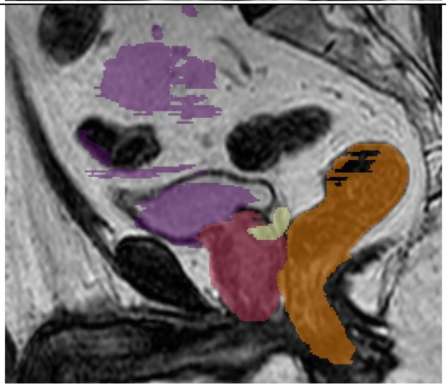	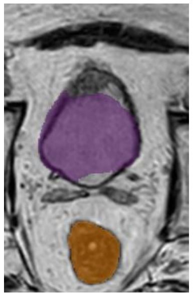	<u>With Augmentation</u><br>Bladder:0.451<br>Prostate:0.801<br>Rectum:0.866<br>SV:0.615<br><u>Without Augmentation</u><br>Bladder: 0.200<br>Prostate: 0.669<br>Rectum:0. 723<br>SV: 0.419

Unannotated & Ground Truth Contour Img	Exp	Without Augmentation		DSC
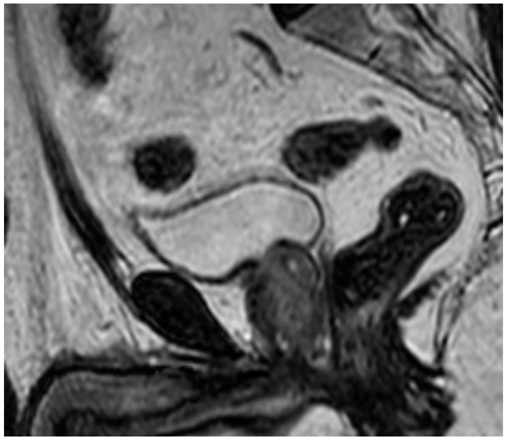	*A* *(100%)*	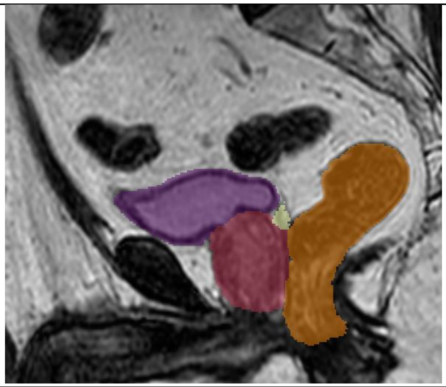	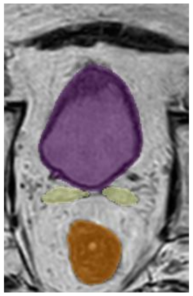	<u>With Augmentation</u><br>Bladder: 0.945<br>Prostate: 0.878<br>Rectum:0.837<br>SV: 0.823<br><u>Without Augmentation</u><br>Bladder: 0.940<br>Prostate: 0.856<br>Rectum:0. 880<br>SV: 0. 837
	D *(50%)*	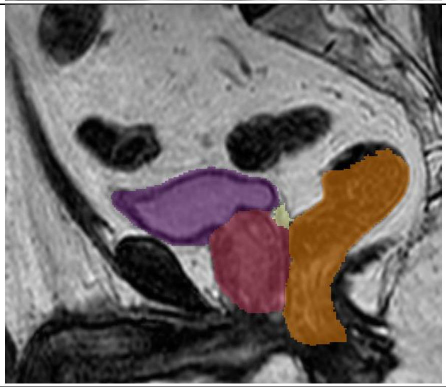	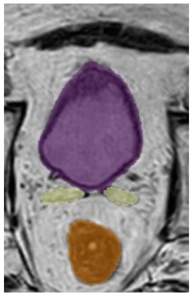	<u>With Augmentation</u><br>Bladder:0.945<br>Prostate:0.862<br>Rectum:0.915<br>SV:0.812<br><u>Without Augmentation</u><br>Bladder: 0. 941<br>Prostate: 0.857<br>Rectum:0.839<br>SV: 0. 812
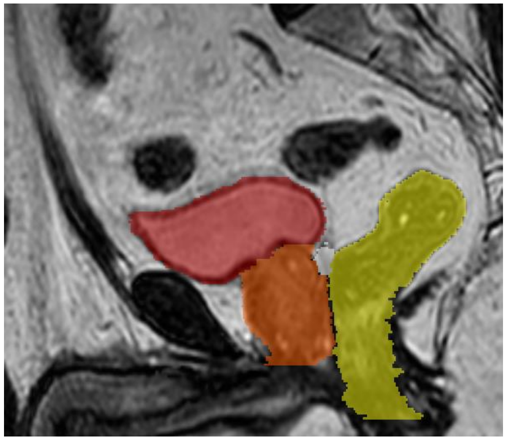	*G* *(12.5%)*	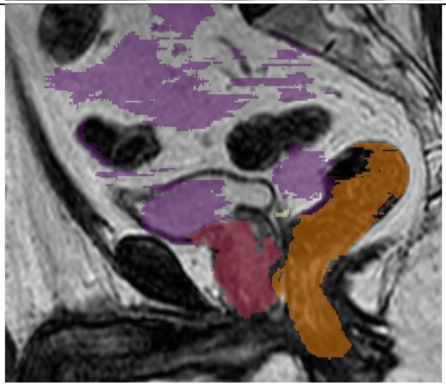	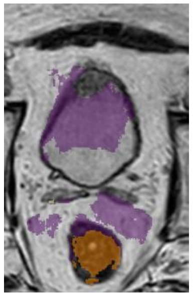	<u>With Augmentation</u><br>Bladder:0.451<br>Prostate:0.801<br>Rectum:0.866<br>SV:0.615<br><u>Without Augmentation</u><br>Bladder: 0.200<br>Prostate: 0.669<br>Rectum:0. 723<br>SV: 0.419

Table 1: Additional Example of Model performance with/out augmentation of the same participant. bladder (purple), prostate (dark pink), rectum (brown), SV (yellow). This participant was selected for having the **worst** average DSC across the four organs in the augmented model inference
